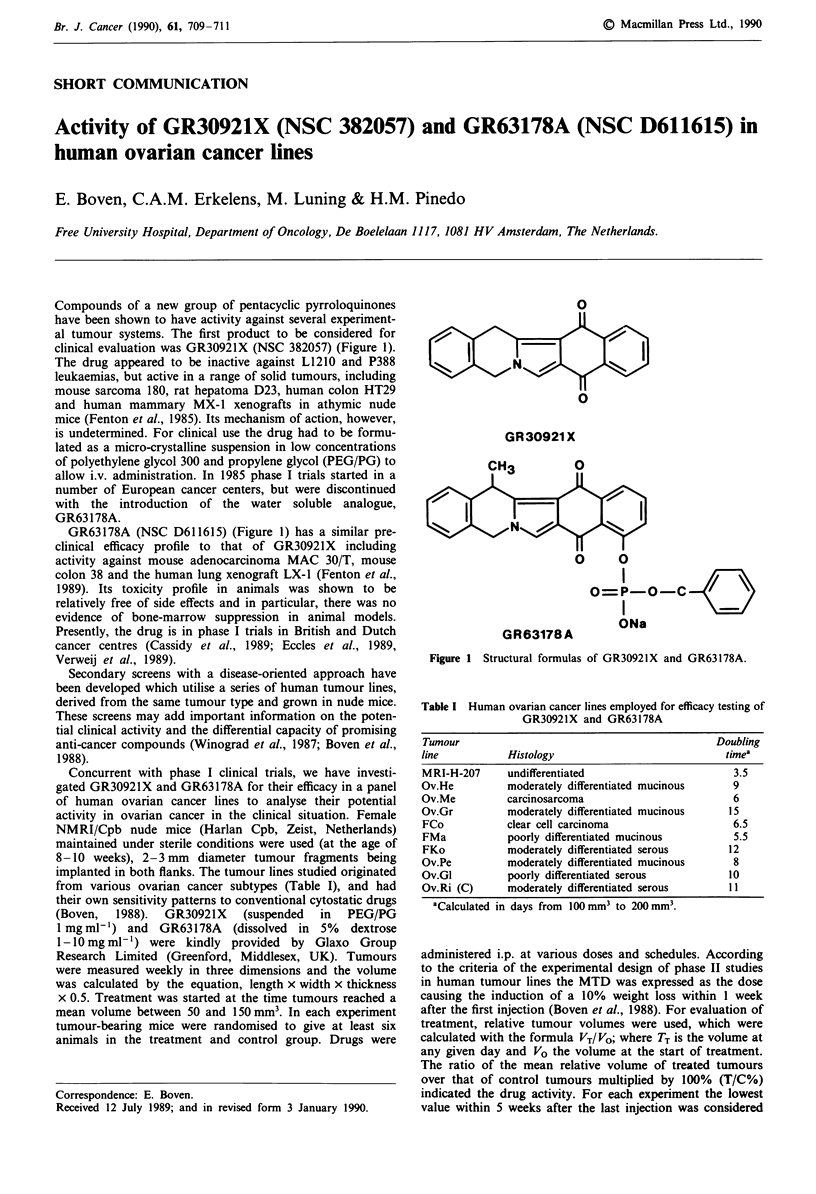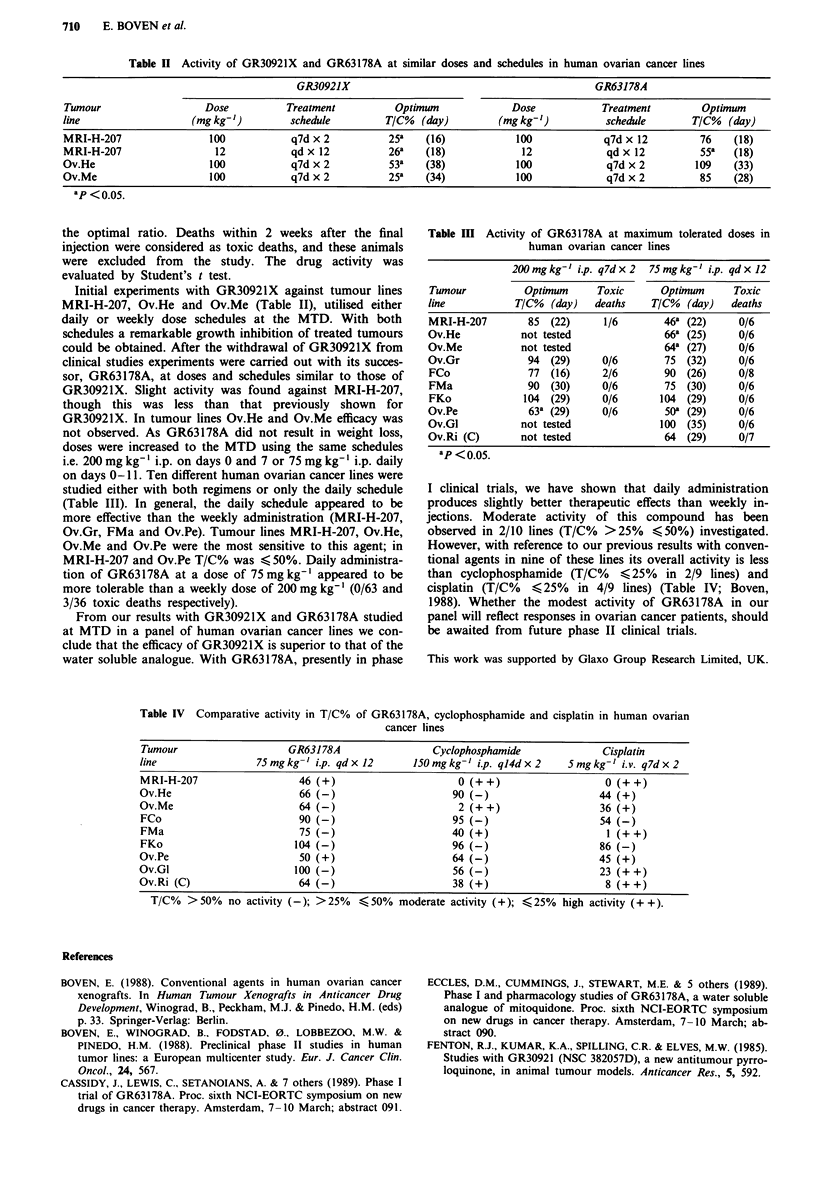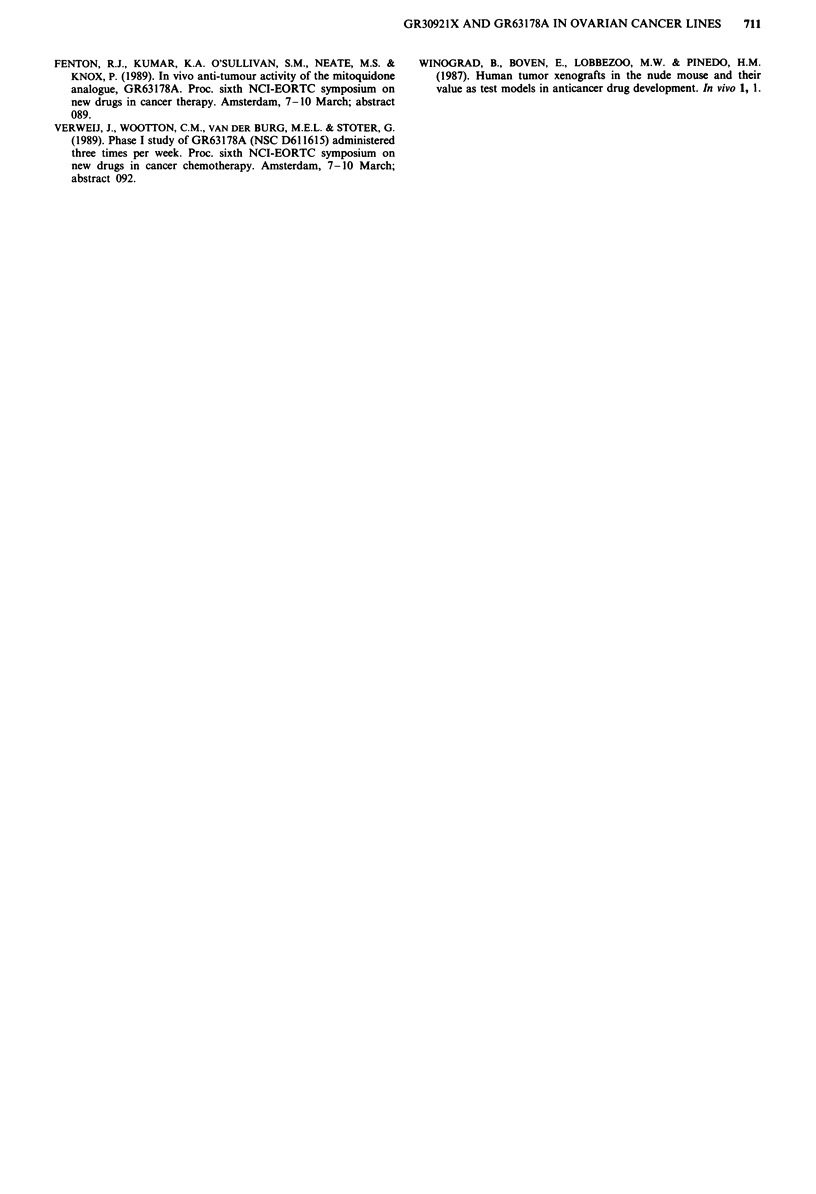# Activity of GR30921X (NSC 382057) and GR63178A (NSC D611615) in human ovarian cancer lines.

**DOI:** 10.1038/bjc.1990.159

**Published:** 1990-05

**Authors:** E. Boven, C. A. Erkelens, M. Luning, H. M. Pinedo

**Affiliations:** Free University Hospital, Department of Oncology, Amsterdam, The Netherlands.


Br. J. Cancer (1990), 61, 709-711

© Macmillan Press Ltd., 1990

SHORT COMMUNICATION

### Activity of GR30921X (NSC 382057) and GR63178A (NSC D611615) in human ovarian cancer lines

E. Boven, C.A.M. Erkelens, M. Luning & H.M. Pinedo

*Free University Hospital, Department of Oncology, De Boelelaan 1117, 1081 HV Amsterdam, The Netherlands.*

Compounds of a new group of pentacyclic pyrroloquinones have been shown to have activity against several experimental tumour systems. The first product to be considered for clinical evaluation was GR30921X (NSC 382057) (Figure 1). The drug appeared to be inactive against L1210 and P388 leukaemias, but active in a range of solid tumours, including mouse sarcoma 180, rat hepatoma D23, human colon HT29 and human mammary MX-1 xenografts in athymic nude mice (Fenton *et al.*, 1985). Its mechanism of action, however, is undetermined. For clinical use the drug had to be formulated as a micro-crystalline suspension in low concentrations of polyethylene glycol 300 and propylene glycol (PEG/PG) to allow i.v. administration. In 1985 phase I trials started in a number of European cancer centers, but were discontinued with the introduction of the water soluble analogue, GR63178A.

GR63178A (NSC D611615) (Figure 1) has a similar preclinical efficacy profile to that of GR30921X including activity against mouse adenocarcinoma MAC 30/T, mouse colon 38 and the human lung xenograft LX-1 (Fenton *et al.*, 1989). Its toxicity profile in animals was shown to be relatively free of side effects and in particular, there was no evidence of bone-marrow suppression in animal models. Presently, the drug is in phase I trials in British and Dutch cancer centres (Cassidy *et al.*, 1989; Eccles *et al.*, 1989, Verweij *et al.*, 1989).

Secondary screens with a disease-oriented approach have been developed which utilise a series of human tumour lines, derived from the same tumour type and grown in nude mice. These screens may add important information on the potential clinical activity and the differential capacity of promising anti-cancer compounds (Winograd *et al.*, 1987; Boven *et al.*, 1988).

Concurrent with phase I clinical trials, we have investigated GR30921X and GR63178A for their efficacy in a panel of human ovarian cancer lines to analyse their potential activity in ovarian cancer in the clinical situation. Female NMRI/Cpb nude mice (Harlan Cpb, Zeist, Netherlands) maintained under sterile conditions were used (at the age of 8-10 weeks), 2-3 mm diameter tumour fragments being implanted in both flanks. The tumour lines studied originated from various ovarian cancer subtypes (Table I), and had their own sensitivity patterns to conventional cytostatic drugs (Boven, 1988). GR30921X (suspended in PEG/PG 1 mg ml$^{-1}$) and GR63178A (dissolved in 5% dextrose 1-10 mg ml$^{-1}$) were kindly provided by Glaxo Group Research Limited (Greenford, Middlesex, UK). Tumours were measured weekly in three dimensions and the volume was calculated by the equation, length × width × thickness × 0.5. Treatment was started at the time tumours reached a mean volume between 50 and 150 mm$^3$. In each experiment tumour-bearing mice were randomised to give at least six animals in the treatment and control group. Drugs were

GR30921X

GR63178A

**Figure 1** Structural formulas of GR30921X and GR63178A.

**Table I** Human ovarian cancer lines employed for efficacy testing of GR30921X and GR63178A

| *Tumour line* | *Histology* | *Doubling time*[a] |
|---|---|---|
| MRI-H-207 | undifferentiated | 3.5 |
| Ov.He | moderately differentiated mucinous | 9 |
| Ov.Me | carcinosarcoma | 6 |
| Ov.Gr | moderately differentiated mucinous | 15 |
| FCo | clear cell carcinoma | 6.5 |
| FMa | poorly differentiated mucinous | 5.5 |
| FKo | moderately differentiated serous | 12 |
| Ov.Pe | moderately differentiated mucinous | 8 |
| Ov.Gl | poorly differentiated serous | 10 |
| Ov.Ri (C) | moderately differentiated serous | 11 |

[a]Calculated in days from 100 mm$^3$ to 200 mm$^3$.

administered i.p. at various doses and schedules. According to the criteria of the experimental design of phase II studies in human tumour lines the MTD was expressed as the dose causing the induction of a 10% weight loss within 1 week after the first injection (Boven *et al.*, 1988). For evaluation of treatment, relative tumour volumes were used, which were calculated with the formula $V_T/V_O$; where $T_T$ is the volume at any given day and $V_O$ the volume at the start of treatment. The ratio of the mean relative volume of treated tumours over that of control tumours multiplied by 100% (T/C%) indicated the drug activity. For each experiment the lowest value within 5 weeks after the last injection was considered

Correspondence: E. Boven.

Received 12 July 1989; and in revised form 3 January 1990.

**Table II** Activity of GR30921X and GR63178A at similar doses and schedules in human ovarian cancer lines

| Tumour line | GR30921X Dose (mg kg$^{-1}$) | Treatment schedule | Optimum T/C% (day) | GR63178A Dose (mg kg$^{-1}$) | Treatment schedule | Optimum T/C% (day) |
|---|---|---|---|---|---|---|
| MRI-H-207 | 100 | q7d × 2 | 25[a] (16) | 100 | q7d × 12 | 76 (18) |
| MRI-H-207 | 12 | qd × 12 | 26[a] (18) | 12 | qd × 12 | 55[a] (18) |
| Ov.He | 100 | q7d × 2 | 53[a] (38) | 100 | q7d × 2 | 109 (33) |
| Ov.Me | 100 | q7d × 2 | 25[a] (34) | 100 | q7d × 2 | 85 (28) |

[a]$P < 0.05$.

the optimal ratio. Deaths within 2 weeks after the final injection were considered as toxic deaths, and these animals were excluded from the study. The drug activity was evaluated by Student's *t* test.

Initial experiments with GR30921X against tumour lines MRI-H-207, Ov.He and Ov.Me (Table II), utilised either daily or weekly dose schedules at the MTD. With both schedules a remarkable growth inhibition of treated tumours could be obtained. After the withdrawal of GR30921X from clinical studies experiments were carried out with its successor, GR63178A, at doses and schedules similar to those of GR30921X. Slight activity was found against MRI-H-207, though this was less than that previously shown for GR30921X. In tumour lines Ov.He and Ov.Me efficacy was not observed. As GR63178A did not result in weight loss, doses were increased to the MTD using the same schedules i.e. 200 mg kg$^{-1}$ i.p. on days 0 and 7 or 75 mg kg$^{-1}$ i.p. daily on days 0–11. Ten different human ovarian cancer lines were studied either with both regimens or only the daily schedule (Table III). In general, the daily schedule appeared to be more effective than the weekly administration (MRI-H-207, Ov.Gr, FMa and Ov.Pe). Tumour lines MRI-H-207, Ov.He, Ov.Me and Ov.Pe were the most sensitive to this agent; in MRI-H-207 and Ov.Pe T/C% was ⩽50%. Daily administration of GR63178A at a dose of 75 mg kg$^{-1}$ appeared to be more tolerable than a weekly dose of 200 mg kg$^{-1}$ (0/63 and 3/36 toxic deaths respectively).

From our results with GR30921X and GR63178A studied at MTD in a panel of human ovarian cancer lines we conclude that the efficacy of GR30921X is superior to that of the water soluble analogue. With GR63178A, presently in phase I clinical trials, we have shown that daily administration produces slightly better therapeutic effects than weekly injections. Moderate activity of this compound has been observed in 2/10 lines (T/C% >25% ⩽50%) investigated. However, with reference to our previous results with conventional agents in nine of these lines its overall activity is less than cyclophosphamide (T/C% ⩽25% in 2/9 lines) and cisplatin (T/C% ⩽25% in 4/9 lines) (Table IV; Boven, 1988). Whether the modest activity of GR63178A in our panel will reflect responses in ovarian cancer patients, should be awaited from future phase II clinical trials.

**Table III** Activity of GR63178A at maximum tolerated doses in human ovarian cancer lines

| Tumour line | 200 mg kg$^{-1}$ i.p. q7d × 2 Optimum T/C% (day) | Toxic deaths | 75 mg kg$^{-1}$ i.p. qd × 12 Optimum T/C% (day) | Toxic deaths |
|---|---|---|---|---|
| MRI-H-207 | 85 (22) | 1/6 | 46[a] (22) | 0/6 |
| Ov.He | not tested | | 66[a] (25) | 0/6 |
| Ov.Me | not tested | | 64[a] (27) | 0/6 |
| Ov.Gr | 94 (29) | 0/6 | 75 (32) | 0/6 |
| FCo | 77 (16) | 2/6 | 90 (26) | 0/8 |
| FMa | 90 (30) | 0/6 | 75 (30) | 0/6 |
| FKo | 104 (29) | 0/6 | 104 (29) | 0/6 |
| Ov.Pe | 63[a] (29) | 0/6 | 50[a] (29) | 0/6 |
| Ov.Gl | not tested | | 100 (35) | 0/6 |
| Ov.Ri (C) | not tested | | 64 (29) | 0/7 |

[a]$P < 0.05$.

This work was supported by Glaxo Group Research Limited, UK.

**Table IV** Comparative activity in T/C% of GR63178A, cyclophosphamide and cisplatin in human ovarian cancer lines

| Tumour line | GR63178A 75 mg kg$^{-1}$ i.p. qd × 12 | Cyclophosphamide 150 mg kg$^{-1}$ i.p. q14d × 2 | Cisplatin 5 mg kg$^{-1}$ i.v. q7d × 2 |
|---|---|---|---|
| MRI-H-207 | 46 (+) | 0 (++) | 0 (++) |
| Ov.He | 66 (−) | 90 (−) | 44 (+) |
| Ov.Me | 64 (−) | 2 (++) | 36 (+) |
| FCo | 90 (−) | 95 (−) | 54 (−) |
| FMa | 75 (−) | 40 (+) | 1 (++) |
| FKo | 104 (−) | 96 (−) | 86 (−) |
| Ov.Pe | 50 (+) | 64 (−) | 45 (+) |
| Ov.Gl | 100 (−) | 56 (−) | 23 (++) |
| Ov.Ri (C) | 64 (−) | 38 (+) | 8 (++) |

T/C% >50% no activity (−); >25% ⩽50% moderate activity (+); ⩽25% high activity (++).

### References

BOVEN, E. (1988). Conventional agents in human ovarian cancer xenografts. In *Human Tumour Xenografts in Anticancer Drug Development*, Winograd, B., Peckham, M.J. & Pinedo, H.M. (eds) p. 33. Springer-Verlag: Berlin.

BOVEN, E., WINOGRAD, B., FODSTAD, Ø., LOBBEZOO, M.W. & PINEDO, H.M. (1988). Preclinical phase II studies in human tumor lines: a European multicenter study. *Eur. J. Cancer Clin. Oncol.*, **24**, 567.

CASSIDY, J., LEWIS, C., SETANOIANS, A. & 7 others (1989). Phase I trial of GR63178A. Proc. sixth NCI-EORTC symposium on new drugs in cancer therapy. Amsterdam, 7–10 March; abstract 091.

ECCLES, D.M., CUMMINGS, J., STEWART, M.E. & 5 others (1989). Phase I and pharmacology studies of GR63178A, a water soluble analogue of mitoquidone. Proc. sixth NCI-EORTC symposium on new drugs in cancer therapy. Amsterdam, 7–10 March; abstract 090.

FENTON, R.J., KUMAR, K.A., SPILLING, C.R. & ELVES, M.W. (1985). Studies with GR30921 (NSC 382057D), a new antitumour pyrroloquinone, in animal tumour models. *Anticancer Res.*, **5**, 592.

FENTON, R.J., KUMAR, K.A. O'SULLIVAN, S.M., NEATE, M.S. & KNOX, P. (1989). In vivo anti-tumour activity of the mitoquidone analogue, GR63178A. Proc. sixth NCI-EORTC symposium on new drugs in cancer therapy. Amsterdam, 7-10 March; abstract 089.

VERWEIJ, J., WOOTTON, C.M., VAN DER BURG, M.E.L. & STOTER, G. (1989). Phase I study of GR63178A (NSC D611615) administered three times per week. Proc. sixth NCI-EORTC symposium on new drugs in cancer chemotherapy. Amsterdam, 7-10 March; abstract 092.

WINOGRAD, B., BOVEN, E., LOBBEZOO, M.W. & PINEDO, H.M. (1987). Human tumor xenografts in the nude mouse and their value as test models in anticancer drug development. *In vivo* **1**, 1.